# The Effect of Glass Fiber-Reinforced Mortars on Physical and Mechanical Performance: An Examination of Length and Ratios

**DOI:** 10.3390/polym18151914

**Published:** 2026-08-04

**Authors:** Suna Cetin, Ahmet Filazi, Reyhan Akat

**Affiliations:** 1 Department of Ceramic, Fine Art Faculty, Cukurova University, Adana 01330, Turkey; cetins@cu.edu.tr; 2 Department of Construction Technology, Kırıkkale Vocational School, Kırıkkale University, Kırıkkale 71450, Turkey; ahmetfilazi@kku.edu.tr; 3 Department of Architecture, Faculty of Engineering and Architecture, Yozgat Bozok University, Yozgat 66200, Turkey

**Keywords:** glass fiber, mortar, length, proportion, physical properties, mechanical properties

## Abstract

This study systematically investigates the combined effects of three different glass fiber lengths (3, 6, and 12 mm) and three different fiber ratios (1%, 2%, and 3%) on the physical, mechanical, and microstructural properties of cement mortars. Glass fibers are known to enhance mortar performance, yet the simultaneous influence of fiber length and proportion on fresh, hardened, durability, and microstructural characteristics has not been comprehensively addressed in a single experimental framework. The parameters investigated include flow diameter, dry unit weight, water absorption, flexural and compressive strength (at 7 and 28 days), ultrasonic pulse velocity, water sorptivity, as well as SEM/EDS and XRD analyses. Results demonstrated that increasing fiber length and content decreased flow diameter and increased porosity within the mortar compositions. At a 3% fiber inclusion, dry unit weight values decreased, with the lowest value (below 2100 kg/m^3^) observed for the 12 mm-3% mixture. Flexural strength results highlighted the critical role of the curing process on fiber-matrix interface development. At 7 days, only 1% fiber content improved flexural strength, while higher contents reduced it. At 28 days, however, increasing fiber content generally enhanced flexural strength, particularly for 6 mm fibers, albeit at the expense of compressive strength. The capillarity test revealed that the optimal fiber proportion was 1%, with mixtures achieving compressive strengths exceeding 50 MPa and capillarity coefficients below 0.10 mm/min^0.5^, while higher fiber contents led to elevated capillarity coefficients. The study concluded that, under the specific conditions tested, the most suitable fiber length is 6 mm, with a recommended fiber content of 1–2%. The 6 mm-1% combination delivered the best overall performance, achieving 60.18 MPa compressive strength, a capillarity coefficient below 0.10 mm/min^0.5^, and enhanced flexural strength. While increasing fiber content improved flexural strength, it reduced compressive strength and increased the capillarity coefficient. These findings underscore the importance of using appropriate fiber proportions to optimize mortar performance and provide practical guidance for designing fiber-reinforced mortars in structural and construction applications.

## 1. Introduction

Concrete remains the primary construction material due to its strength, affordability, and versatility. However, concrete’s inherent low tensile strength, limited energy absorption, and minimal flexural toughness often restrict its effectiveness in structural applications. These limitations typically manifest as cracking due to drying shrinkage and reduced long-term durability [1,2,3,4].

To address these issues, fibers are often added to enhance concrete’s mechanical properties and durability. Fibers randomly distributed in the matrix resist crack propagation and widening, thereby reducing brittleness and improving post-cracking ductility, strength, and energy absorption capacity under both static and impact loads [5,6,7]. Extensive research is currently being conducted to assess and identify the advantages of fiber–reinforced concrete [8,9,10,11]. A wide range of fibers, including steel [12], carbon [13], glass [14], polypropylene [15], and polyolefin [16], are utilized in concrete production.

Among these, glass fibers are particularly attractive due to their combination of low cost and high tensile strength [17,18]. The growth of the polymer composites market is supported by the widespread use of low-cost glass fibers, which are used in over 95% of reinforced composite products. Glass fiber-reinforced concretes exhibit higher tensile strength and enhanced resistance to brittleness compared to conventional concrete [19]. Additionally, this material can control cracking caused by drying shrinkage and improve the mechanical properties of concrete [20].

Research has shown that incorporating glass fibers substantially enhances flexural and compressive strength [21]. However, overall effectiveness is heavily governed by fiber parameters, such as length and dosage [22,23,24]. For example, significant improvements in recycled glass fiberreinforced mortars have been reported, with compressive strength increases of 13.6% to 31.5% and splitting tensile strength gains up to 97.6% [25], alongside enhanced 28-day performance [26].

Recent studies have further explored these parameters across various matrix types. Wu et al. [27] investigated the effect of fiber content on alkali-resistant glass fiber-reinforced concrete, finding that while moderate fiber ratios improve mechanical and durability properties, excessive amounts cause fiber clustering. Ali and Qureshi [28] reported similar performance trends in recycled aggregate concrete reinforced with glass fibers. Simões et al. [29] demonstrated that higher micro-fiber dosages could increase matrix porosity, whereas Mazloom et al. [30] highlighted the trade-offs between capillary absorption and fracture energy in macro-fiber concrete. Furthermore, Abdi Moghadam and Izadifard [31] evaluated high-temperature resilience, and Bencardino et al. [32] demonstrated the viability of textile-reinforced sustainable alkali-activated mortars for structural applications.

Despite extensive research on the short-term mechanical performance of glass fiber-reinforced mortars, the coupled effects of structural fiber parameters and their long-term performance remain critical areas requiring systematic investigation. To address this gap, this study investigates the performance of glass fiber-reinforced mortars incorporating fiber lengths of 3, 6, and 12 mm at dosages of 1%, 2%, and 3% by weight. The primary novelty lies in an integrated evaluation of the combined effects of fiber length and content on both macroscopic mechanical behavior and microstructural mechanisms. To this end, the following hypotheses were tested: (i) increasing fiber length and content reduces workability while increasing porosity; (ii) an optimum fiber content exists that maximizes compressive strength; and (iii) a 6 mm fiber length provides the optimal balance between mechanical performance and durability. Ultimately, these findings aim to establish practical design guidelines for optimizing glass fiber reinforcement in cementitious mortars requiring enhanced crack control and durability.

## 2. Materials and Methods

### 2.1. Materials

#### 2.1.1. Cement

In this study, Portland cement (PC) of type CEM I 42.5 R, which corresponds to ASTM C150 Type I cement [33], was used. The specific gravity and Blaine fineness of the cement, determined in the Building Materials Laboratory of the Civil Engineering Department at Kırıkkale University, were found to be 3366 cm^2^/g. The physical and chemical properties of the cement used are given in Table 1.

#### 2.1.2. Standard Sand

The standard sand utilized in this study is the CEN reference sand, manufactured at the Limak Trakya Cement factory, as specified in [34] standards. The grain size distribution of the reference sand is detailed in Table 2.

#### 2.1.3. Glass Fiber

Glass fibers are thin fibers obtained by drawing out glass or through special production methods. These fibers are commonly used in many industrial applications due to their thermal, chemical, and mechanical durability. Glass fibers are also known as fiberglass or glass filaments. They typically have a diameter in the micron scale and can be produced in various lengths. Glass fibers find applications in a wide range of fields, including reinforcement of composite materials, production of insulation materials, filtration systems, electronic and electrotechnical applications.

**Figure 1:**
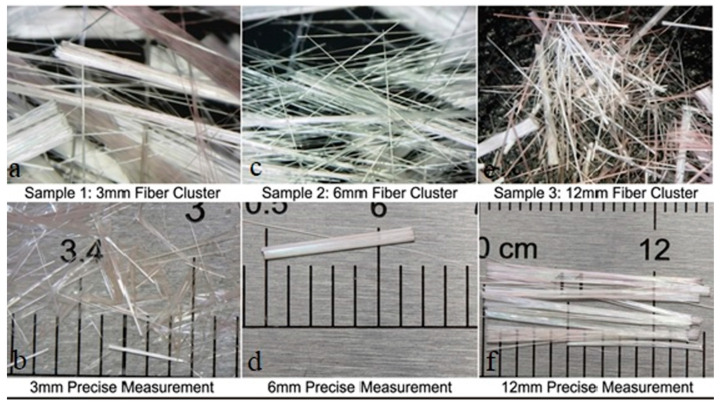
Microscopic characterization and precise length measurements of glass fibers: (**a**,**b**) 3 mm, (**c**,**d**) 6 mm, and (**e**,**f**) 12 mm fiber clusters and individual lengths, respectively.

Water-reducing additives were notably absent in the preparation of the mixtures, with solely drinking water utilized. Glass fibers were incorporated at lengths of 3, 6, and 12 mm. Figure 1 illustrates microscopic images of the glass fibers.

In Table 3, glass fiber is generally a form of glass in the form of long thin fibers. The chemical and physical properties of glass fibers may differ from the general properties of glass.

### 2.2. Sample Preparation

For the mixture preparation, a water-to-cement (w/c) ratio of 0.50 and an aggregate-to-cement (a/c) ratio of 3 were selected. This means that for every unit of cement, half a unit of water is used and the aggregate quantity is three times that of cement. The quantity of glass fibers was determined based on the cement content. Fibers were introduced into the mix at rates of 1%, 2%, and 3% relative to the weight of the cement. Table 4 outlines the ratios of the mixtures and the quantities of materials utilized. The addition of fibers, varying in lengths and proportions, was carried out across 10 mixtures. These included 9 distinct fiber mixtures and 1 reference mixture devoid of fibers. Glass fibers were incorporated into the mixtures as the final step. Following thorough blending of aggregate, water, and cement, fibers were added to the mix. The fiber mixture underwent stirring for 1 min at low speed and 1 min at high speed to ensure homogeneity.

### 2.3. Experimental Methods

#### 2.3.1. Flow Diameter

**Figure 2:**
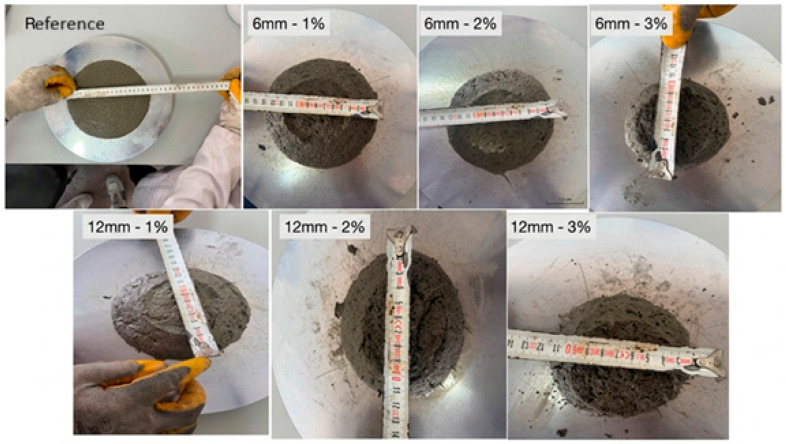
Representative photographs of the flow diameters for control and fiber-reinforced cement mortars.

The flow diameter of the fresh mortar mixtures was measured using the method specified in ASTM C1437 [35], which is standardly used for hydraulic cement mortars. The fresh mixture was cast into the standard flow mold, which was then lifted vertically to allow free casting spread on the flow table. The measured average spread diameter serves as a direct indicator of the material’s fluidity. Figure 2 displays the fresh state characteristics of selected mortar samples, offering a visual contrast between satisfactory and unsatisfactory flowability within the tested mixtures.

#### 2.3.2. Dry Bulk Density Test Method

The unit weight of each sample was determined following a standardized procedure. Initially, cubic samples with dimensions of 50 × 50 × 50 mm were prepared from various mortar mixes, adhering to the guidelines outlined in [36] standards. Subsequently, these samples underwent a curing process for a period of 28 days under controlled laboratory conditions at 20 ± 2 °C temperature and ≥95% relative humidity, in accordance with TS EN 196-1 [33] standards, to ensure adequate hardening. Upon completion of the curing period, each sample was carefully weighed using a precision balance to obtain its weight in grams (g). The unit weight of each sample was then calculated by dividing its weight by its volume. The resulting unit weight values were recorded systematically for subsequent analysis and comparison purposes. This comprehensive procedure ensured accurate determination of the unit weight of the mortar samples, providing valuable insights into their physical properties and characteristics.

#### 2.3.3. Water Absorption Percentage

The test was conducted according to ASTM C1403-13 [37] after 28 days of curing to assess their long-term durability performance. In the research, three companion samples for each mixture were oven-dried at 105 °C until reaching a constant mass. Subsequently, the samples were left in the laboratory environment to cool down. Afterwards, the samples were immersed in water for 24 h to measure their wet weight. The water absorption percentage Wa% was then calculated according to Equation (1):

(1) $$Wa\%=\frac{w2-w1}{w1}\times 100$$

where Wa indicates water absorption percentage; w1 is the dry weights; and w2 is the saturated-wet weights of the samples.

#### 2.3.4. Flexural and Compressive Strengths

**Figure 3:**
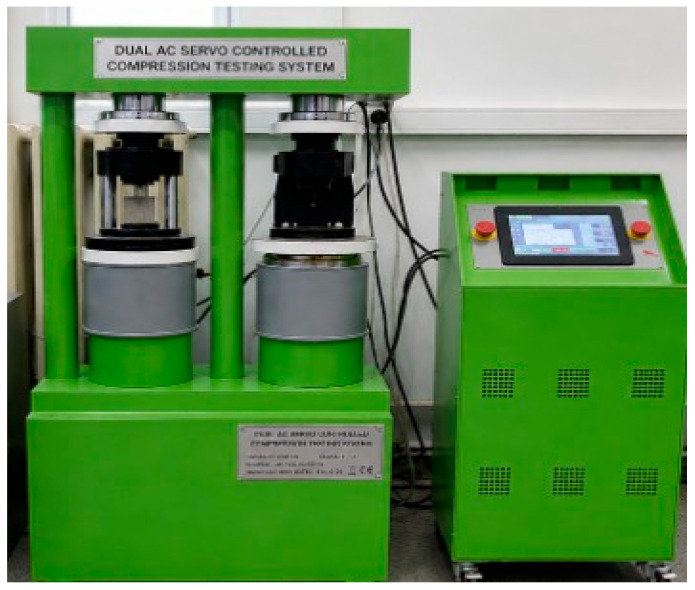
Flexural and compressive strength testing equipment along with cured test specimens.

The compressive and flexural strengths of the mortar specimens were determined in accordance with ASTM C349-24 [38] and ASTM C348-21 [39], respectively. Prismatic specimens measuring 40 × 40 × 160 mm were prepared, cured for 7 and 28 days, and subsequently tested for mechanical strength, as shown in Figure 3.

#### 2.3.5. Ultrasonic Pulse Velocity Experiment

Following a 28-day curing period, the mortar samples were examined by measuring sound transit times (t, μs) using an ultrasonic device in accordance with ASTM C 597 [40]. The ultrasonic pulse velocity (UPV) values are categorized to evaluate the quality according to ASTM C597 [41]: values above 4.5 km/s indicate “excellent” quality, values between 3.5–4.5 km/s indicate “good” quality, values between 3.0–3.5 km/s indicate “fair” quality, and values below 3.0 km/s indicate “poor” quality.

#### 2.3.6. Water-Sorptivity Tests

**Figure 4:**
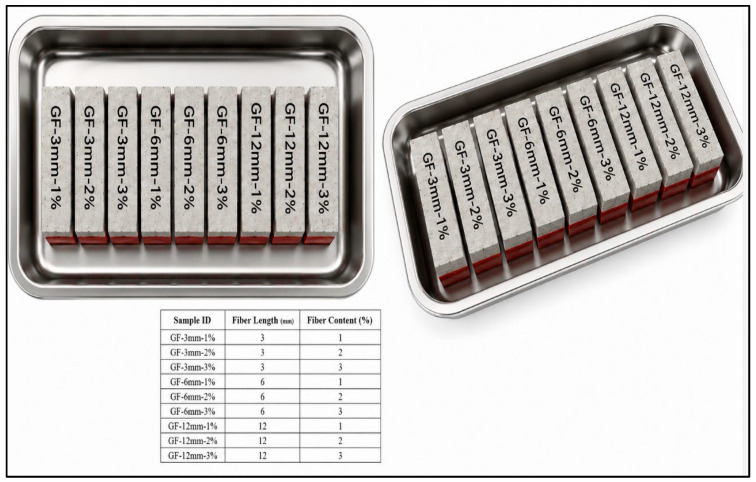
Capillary water absorption (sorptivity) test assembly and prismatic specimen configurations (40 × 40 × 160 mm) for cement mortars.

Water sorptivity was determined according to ASTM C1585 [42], on prismatic specimens with dimensions of 40 ×40 × 160 mm after 28 days of curing (Figure 4). Although ASTM C1585 typically recommends disc specimens (diameter 100 mm, thickness 50 mm) or prismatic specimens with specified cross-sections, the prismatic geometry (40 × 40 mm cross-section) used in this study is acceptable for comparative analysis provided that one-dimensional water ingress is ensured. Prior to testing, the specimens were oven-dried at 50 ± 2 °C until constant mass was achieved (approximately 72 h). After cooling to room temperature in a sealed container, the lateral surfaces of the specimens were sealed with an impermeable epoxy resin to ensure one-dimensional water ingress through the bottom surface only, as required by ASTM C1585 [42]. The exposed bottom surface (40 × 40 mm) was then immersed in distilled water at a depth of approximately 3–5 mm to maintain consistent contact with water. The mass increase of each specimen was recorded at regular time intervals (1, 5, 10, 20, 30, 60, 120, 180, 240, and 1440 min), in accordance with ASTM C1585 [42]. The cumulative water absorption per unit area (mm) was plotted against the square root of time (min^0.5^), and the sorptivity coefficient (mm/min^0.5^) was calculated as the slope of the initial linear portion of the curve (typically between 1 and 60 min, as specified in ASTM C1585) [42]. Three specimens were tested for each mixture, and the average values are reported.

#### 2.3.7. Scanning Electron Microscopy (SEM) and EDS Analysis

The microstructural surface morphology and elemental composition of the specimens were characterized using a high-resolution Quanta 650 Field Emission Scanning Electron Microscope (FE-SEM, Thermo Fisher Scientific, Eindhoven, The Netherlands) equipped with an Energy Dispersive X-ray Spectroscopy (EDS) system. The analysis was performed on specimens that had completed a 28-day curing period. The instrument was operated at an acceleration voltage of 20 kV to ensure optimal imaging and analytical precision. Due to the non-conductive nature of the mortar matrices, the specimens were sputter-coated with a thin conductive layer of gold (Au) at a deposition rate of approximately 2 Å/s prior to the analysis to eliminate surface charging effects. A Secondary Electron (SE) detector was utilized to evaluate the surface topography and the fiber-matrix interfacial transition zone (ITZ), while EDS point and area analyses were employed to evaluate local elemental chemistry.

It should be noted that EDS microanalysis on rough, fractured surfaces is inherently semi-quantitative and reflects the local chemical composition of specific micro-areas rather than the overall bulk matrix. Therefore, the obtained elemental ratios (e.g., Ca/Si) characterize localized chemical micro-environments-such as localised crystalline portlandite deposits-and should not be interpreted as the average bulk composition of the mortar matrix.

#### 2.3.8. X-Ray Diffraction (XRD) Analysis

X-ray diffraction (PANalytical B.V., Almelo, The Netherlands) (XRD) analysis was performed on selected mortar samples to identify and semi-quantitatively compare the crystalline phases, particularly portlandite (Ca(OH)_2_), calcite (CaCO_3_), and unhydrated clinker phases (C_3_S, C_2_S). Representative fragments were collected from the core of 28-day cured specimens, ground to a fine powder (<45 µm), and analyzed using a Panalytical Empyrean with Cu-Kα radiation (λ = 1.5406 Å) operating at 40 kV and 40 mA. Scans were conducted over a 2θ range of 10–70° with a step size of 0.02° and a counting time of 1 s per step. Phase identification was performed using reference to ICDD PDF-2 database. Relative phase changes among mixtures were evaluated qualitatively based on characteristic peak intensity comparisons; full quantitative phase was not performed in this study.

## 3. Results and Discussion

### 3.1. Properties of Fresh Mortar

**Figure 5:**
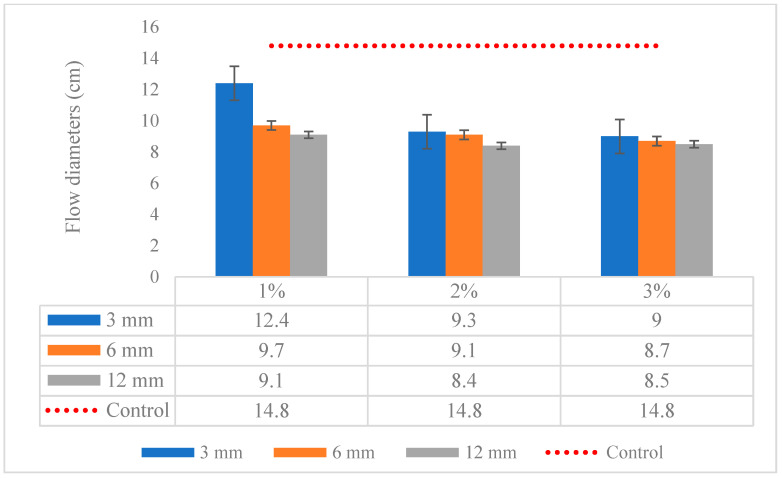
Effect of different fiber lengths and fiber ratios on the flow diameters of the mortar mixtures.

Figure 5 presents a detailed analysis of how the workability characteristics of mortar mixtures are influenced by both glass fiber length and content. The study reveals that as the length and content of glass fibers increase, there is a general decrease in flow diameters. Notably, a significant reduction in flow diameters is observed, particularly when the glass fiber content reaches 2% and 3%. Moreover, as the length of glass fibers increases, mortar samples containing 1% fiber content demonstrate the most substantial decline in workability. Samples using 2% and 3% glass fiber content exhibited similar characteristics in terms of flow diameters, but the use of 6 mm length or 3% glass fiber negatively affected workability. The addition of glass fibers reduced the fluidity of the mixture, with the most pronounced decrease observed for the 12 mm-3% mixture (8.5 cm), representing a 42.6% reduction compared to the control mixture (14.8 cm). This study underscores the considerable impact of fibers on the workability properties of mortar, illustrating a decrease in flow diameter as both fiber content and length increase. This observed decline in workability properties aligns with findings reported in existing literature.

### 3.2. Dry Bulk Density Results

**Figure 6:**
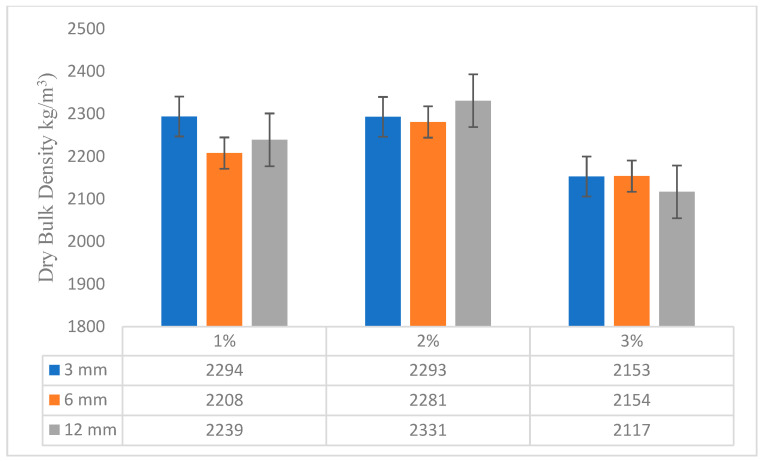
Dry bulk density of mortar mixtures with different fiber lengths and ratios.

According to the data presented in Figure 6, different effects of fiber length on the density values of mortar mixtures are observed. According to the data presented in Figure 6, the effect of fiber length on the dry bulk density of mortar mixtures is strongly dependent on the fiber content. At 1% fiber content, mixtures containing 3 mm fibers exhibited the highest density (approximately 2180 kg/m^3^), followed by those with 6 mm and 12 mm fibers, indicating that shorter fibers can enhance matrix packing at low dosages. However, at 2% fiber content, this trend shifted, and the 12 mm mixture showed comparable or slightly higher density than the 3 mm mixture, suggesting that packing efficiency varies with fiber length at moderate contents. At 3% fiber content, a general decrease in density was observed for all mixtures due to increased air void formation and matrix discontinuity. Specifically, among the 3% fiber content mixtures, the dry bulk density values were approximately 2150 kg/m^3^ (3 mm-3%), 2110 kg/m^3^ (6 mm-3%), and 2080 kg/m^3^ (12 mm-3%). Only the 12 mm-3% mixture exhibited a value below the 2100 kg/m^3^ threshold, demonstrating that the combination of longer fibers and high dosages promotes notable void entrapping within the mortar matrix.

In conclusion, fiber length and fiber content exhibit a coupled influence on the dry bulk density of cementitious mortars. While shorter fibers (3 mm) promote better matrix packing and higher density at low dosages (1%), longer fibers (12 mm) at high dosages (3%) induce microstructural discontinuity and increase air void entrapping, leading to a noticeable drop in density (below 2100 kg/m^3^). These findings underscore the critical importance of optimizing both fiber length and dosage to balance fresh-state workability with hardened physical properties in mortar design.

### 3.3. Water Absorption Results

**Figure 7:**
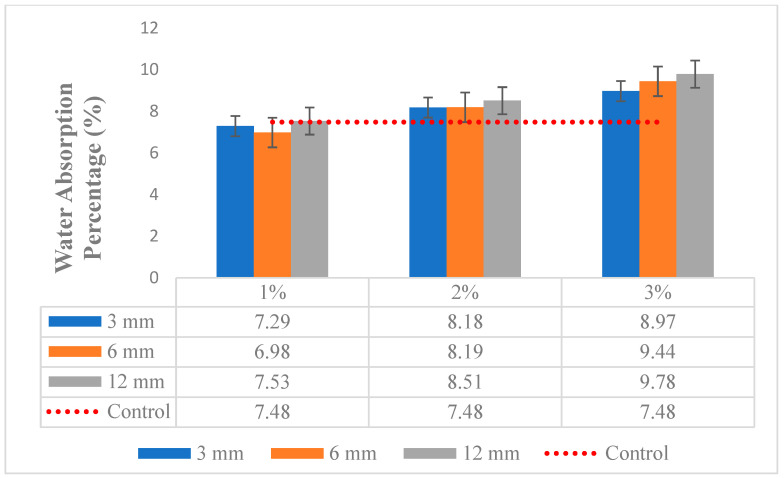
Water absorption percentage of mortar specimens with different fiber lengths and ratios.

The effect of fiber length and content on the water absorption percentage of mortar mixtures is presented in Figure 7. For all fiber lengths, water absorption increased with increasing fiber content. In mixtures containing 3 mm fibers, for example, water absorption rose from 7.29% at 1% fiber content to 8.97% at 3% fiber content. Similar increasing trends were observed for 6 mm and 12 mm fibers. When comparing different fiber lengths at the same fiber content, mixtures with 6 mm and 12 mm fibers generally exhibited higher water absorption values than those with 3 mm fibers. This indicates that longer fibers create additional void spaces within the matrix, likely due to increased fiber entanglement, poor dispersion, and inadequate matrix penetration around longer fibers.

A study examining the impact of various fibers, including glass fiber (GF), polypropylene, and steel fibers, on the mechanical properties and porosity of mortars revealed that at higher dosages (2% volume fractions), both steel fibers (SFs) and glass fibers (GFs) increased the porosity of the mortar compared to the unreinforced mortar [29]. Incorporating glass fibers influences matrix porosity and tortuosity. At lower dosages, fine fiber dispersion restricts capillary network connectivity, whereas excessive fiber contents (>2%) tend to increase interfacial micro-voids, thereby slightly elevating overall water absorption percentages [19]. In this context, water absorption percentage values of mortar mixtures are affected by both fiber length and ratio. Specifically, an increase in fiber ratio and length has been noted to lead to higher porosity values. The length and ratio of fibers employed in mortar mixtures play a crucial role in determining the mixture’s porosity, emphasizing the importance of accounting for these factors in mortar mixture design [29,43].

### 3.4. Mechanical Properties of Mortars

**Figure 8:**
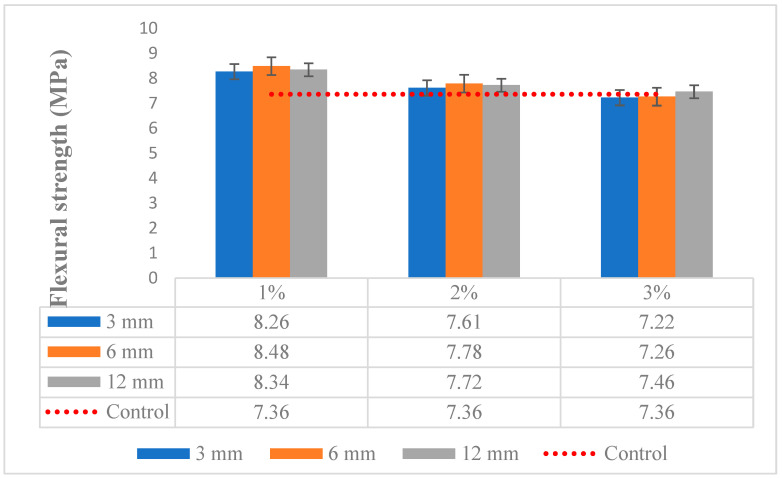
Seven-day flexural strength of mortar specimens with different fiber lengths and ratios.

**Figure 9:**
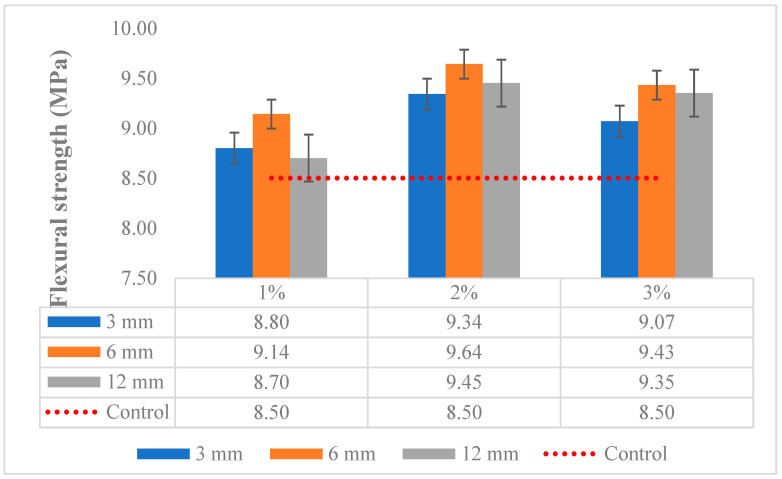
Twenty-eight-day flexural strength of mortar specimens with different fiber lengths and ratios.

Upon examining the results of the flexural strength of mortars for 7 and 28 days (Figure 8 and Figure 9), a notable observation emerges: the effect of fiber content on flexural strength differs considerably between early and later ages. At 7 days, increasing fiber content leads to a decrease in flexural strength, while at 28 days, the interface continues to develop over time, and early-age defects (such as voids and weak interfaces) are partially compensated by ongoing hydration products, including C-S-H, portlandite, and calcite precipitation, which densify the matrix around the fibers.

To further explain the superior performance of the 6 mm fibers over other lengths, the concept of critical fiber length (Lc) was evaluated based on the Kelly–Tyson micromechanical model (Lc = σf.d/2τ). According to this model, Lc depends on the fiber tensile strength (σf), fiber diameter (d), and the interfacial bond strength (τ) between the fiber and the matrix. Using the specified tensile strength (1700 MPa) and diameter (13 µm) from Table 3, along with representative interfacial shear strength values reported in the literature (τ ≈ 3–5 MPa), the estimated critical fiber length ranges between approximately 4.4 and 5.5 mm [27]. Consequently, the 6 mm fibers lie slightly above the calculated critical threshold, providing the optimal length required for efficient stress transfer across micro-cracks without premature pull-out. In contrast, the 3 mm fibers fall below this critical length (<Lc) and cannot fully utilize their tensile load-bearing capacity before being pulled out. Conversely, while the 12 mm fibers exceed Lc, their high aspect ratio makes them susceptible to fiber entanglement and non-uniform dispersion during mixing, introducing macroscopic defects that compromise reinforcement efficiency.

Based on the data presented in Figure 8, the 7-day flexural strength of mortar mixtures is significantly influenced by both fiber length and content. The reference mixture achieved a flexural strength of 7.36 MPa. At a fiber content of 1%, all mixtures exhibited higher flexural strength than the reference, regardless of fiber length. The highest value was observed for the 6 mm-1% mixture (8.48 MPa), followed by the 12 mm-1% (8.34 MPa) and 3 mm-1% (8.26 MPa) mixtures. This indicates that a low fiber content (1%) improves early-age flexural strength, likely due to effective fiber-matrix bonding and stress transfer.

However, as the fiber content increased to 2% and 3%, a clear decreasing trend in flexural strength was observed for all fiber lengths. For example, in mixtures with 3 mm fibers, strength dropped from 8.26 MPa (1%) to 7.61 MPa (2%) and further to 7.22 MPa (3%). Similar reductions were noted for 6 mm and 12 mm fibers. Despite these decreases, most mixtures with 2% and 3% fiber content still maintained flexural strength values close to or slightly above the reference level, with the exception of the 3 mm-3% and 6 mm -3% mixtures, which fell marginally below the reference.

In summary, while a 1% fiber content enhances 7-day flexural strength, increasing the fiber ratio to 2% or 3% leads to a progressive reduction in strength across all fiber lengths. This trend is attributed to reduced workability and increased fiber agglomeration at higher fiber contents, which negatively affect early-age mechanical performance.

Examining the flexural strengths of mortar mixtures with different fiber lengths and ratios at 28 days (Figure 9) reveals several key observations. Overall, as the fiber ratio increases, flexural strength tends to increase; however, the effect varies depending on fiber length.

For mixtures with 3 mm fibers, flexural strength increased by 3.5% at 1% fiber ratio, with further increases of 9.9% and 6.7% at 2% and 3% ratios, respectively, compared to the reference (8.50 MPa). For 6 mm fibers, flexural strength increased by 7.5% at 1% ratio, and by 13.4% and 10.9% at 2% and 3% ratios, respectively. For 12 mm fibers, the increases were 2.4% at 1%, 11.2% at 2%, and 10.0% at 3%.

These findings confirm that increasing fiber content generally enhances flexural strength. However, the rate of improvement diminishes at higher ratios, and for 3 mm and 6 mm fibers, a slight decrease was observed at 3% compared to 2%, suggesting that excessive fiber content may negatively affect performance. The 6 mm-2% mixture exhibited the highest flexural strength (9.64 MPa), representing a 13.4% increase over the reference.

In conclusion, while higher fiber ratios improve flexural strength, the benefits plateau beyond an optimum level. Therefore, careful selection of both fiber length and ratio is essential to achieve optimal mechanical performance in mortar mixtures.

**Figure 10:**
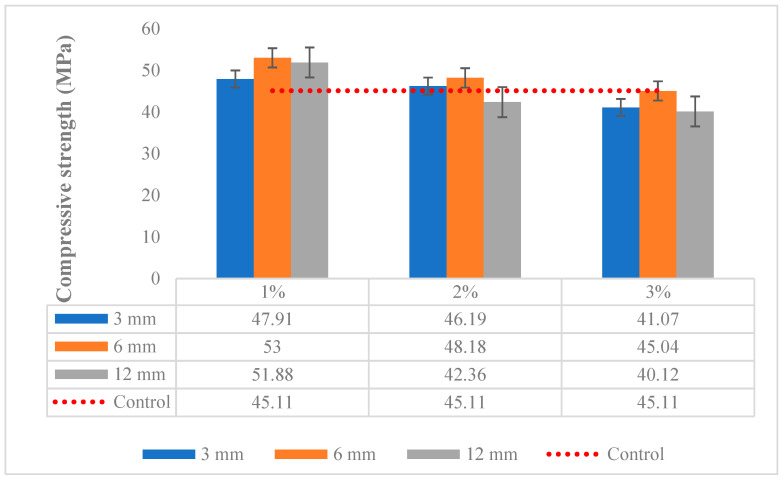
Seven-day compressive strength of mortar specimens with different fiber lengths and ratios.

Based on the data presented in Figure 10, the 7-day compressive strength of mortar mixtures is significantly influenced by both fiber length and content. The reference mixture achieved a compressive strength of 45.11 MPa.

At 1% fiber content, all mixtures exhibited higher compressive strength than the reference. The highest value was observed for the 6 mm-1% mixture (53.00 MPa), representing a 17.5% increase over the reference. The 12 mm-1% mixture (51.88 MPa) and 3 mm -1% mixture (47.91 MPa) also showed improvements of 15.0% and 6.2%, respectively. This indicates that a low fiber content (1%) can enhance early-age compressive strength, particularly for 6 mm fibers.

However, as the fiber content increased to 2% and 3%, a clear decreasing trend in compressive strength was observed for all fiber lengths. For 6 mm fibers, strength dropped from 53.00 MPa (1%) to 48.18 MPa (2%) and further to 45.04 MPa (3%). Notably, the 6 mm-2% mixture (48.18 MPa) still remained above the reference value, while the 6 mm-3% mixture (45.04 MPa) fell slightly below it. For 3 mm fibers, strength decreased from 47.91 MPa (1%) to 46.19 MPa (2%) and 41.07 MPa (3%), with the 3 mm-3% mixture falling significantly below the reference. For 12 mm fibers, strength dropped from 51.88 MPa (1%) to 42.36 MPa (2%) and 40.12 MPa (3%), both 2% and 3% mixtures falling below the reference.

In summary, 1% fiber content improves 7-day compressive strength regardless of fiber length, with 6 mm fibers providing the best performance. Increasing the fiber ratio to 2% or 3% leads to a progressive reduction in strength, although 6 mm fibers at 2% content still maintain acceptable strength above the reference level. The most severe reductions were observed for 12 mm fibers at 2% and 3% content, as well as 3 mm fibers at 3% content.

**Figure 11:**
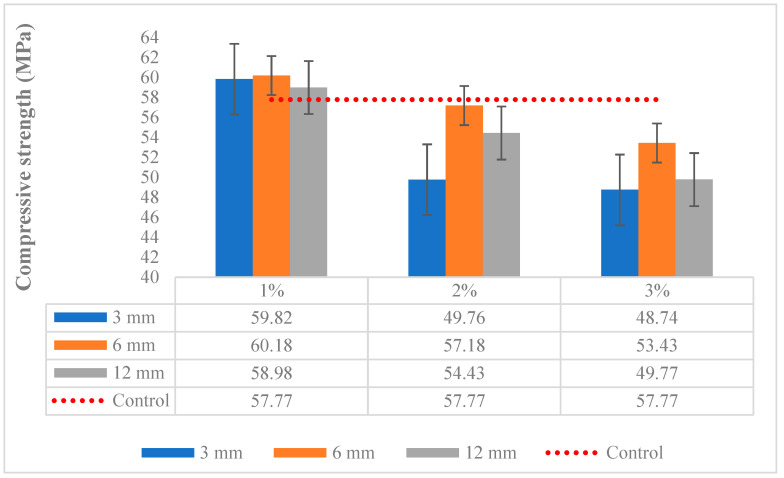
Twenty-eight-day compressive strength of mortar specimens with different fiber lengths and ratios.

Analyzing the data presented in Figure 11, the effect of glass fiber content on the 28-day compressive strength is highly dependent on fiber length.

For mixtures with 3 mm fibers, compressive strength decreased markedly from 59.82 MPa (1%) to 49.76 MPa (2%) and 48.74 MPa (3%), representing reductions of 16.8% and 18.5%, respectively, compared to the 1% content.

For 12 mm fibers, a similar decreasing trend was observed: 58.98 MPa (1%) to 54.43 MPa (2%) and 49.77 MPa (3%). The reductions were 7.7% and 15.6%, respectively, compared to the 1% content.

Interestingly, for 6 mm fibers, the strength remained relatively stable at 1% and 2% (57.18 MPa), showing only a 1.0% reduction compared to the reference, before dropping to 53.43 MPa at 3% (an 11.2% reduction from 1% content). Notably, the 6 mm-2% mixture remained comparable to the reference mixture, indicating that this combination can maintain compressive strength equivalent to the unreinforced mortar while providing additional flexural benefits. This suggests that 6 mm fibers at a 2% dosage can maintain compressive strength comparable to the reference, likely due to better fiber dispersion and more effective stress transfer at this length.

In contrast, 3 mm fibers appear too short to provide effective bridging, while 12 mm fibers tend to entangle and create defects even at moderate contents. Overall, the results indicate that the optimum fiber content for compressive strength is 1% for all lengths, but 6 mm fibers at 2% content still provide acceptable performance.

### 3.5. Sorptivity Properties of Mortars

**Figure 12:**
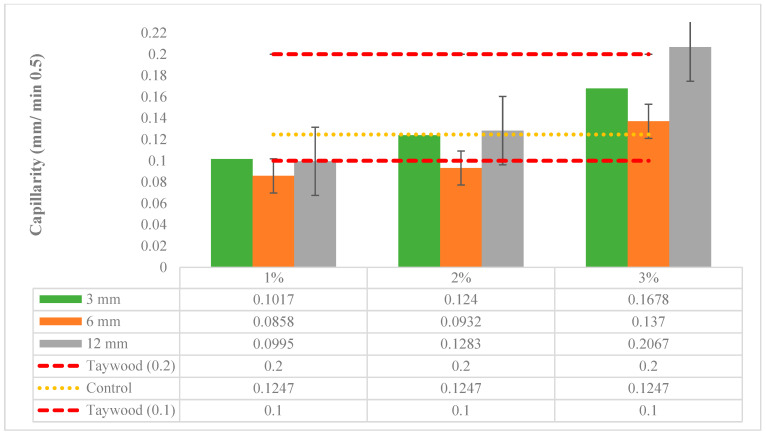
Sorptivity properties of mortar specimens with different fiber lengths and ratios.

Based on the analysis conducted using the data presented in Figure 12, it has been observed that different fiber lengths and ratios have notable effects on the capillary water absorption of mortar mixtures. The quality classification for cement-based composites proposed by Taywood [44] is an important assessment criterion based on the materials’ capillary water absorption properties. This classification system proves highly valuable in assessing the materials’ resistance to water. For instance, if the capillarity coefficient measures below 0.10 mm/min^0.5, the material is categorized as “good” and demonstrates strong resistance to water. Conversely, if the capillarity coefficient exceeds 0.2 mm/min^0.5, the material is classified as “poor” and exhibits weaker resistance to water.

According to our data, an increase in fiber length in cement-based composites leads to an increase in capillary water absorption. Particularly, the highest capillary water absorption is achieved with a 12 mm fiber length at 3% content (0.2067 mm/min^0.5^), which exceeds the “poor” threshold (>0.20). This indicates that longer fibers facilitate the movement of water within the composite material’s internal structure, thus increasing the water absorption.

However, our analysis determined that at 1% fiber content, the capillarity coefficients remain below or near 0.10 mm/min^0.5^ for all fiber lengths (3 mm: 0.1017, 6 mm: 0.0858, 12 mm: 0.0995), indicating “good” resistance to water per Taywood’s classification. For 2% and 3% fiber contents, the capillary water absorption increases to “moderate” levels, suggesting that cement-based composites with higher fiber ratios exhibit reduced resistance to water.

In conclusion, fiber length and ratio have a considerable impact on the capillary water absorption properties of cement-based composites. Taywood’s [44] classification provides an important guide for evaluating the materials’ resistance to water.

Recent studies have highlighted the significant impact of fiber ratios on the properties of mortar mixtures. For instance, glass fibers were investigated for their use in cement-based mortars, and it was observed that an increase in fiber ratios led to a rise in the capillarity coefficients of the mixtures. The tendency of fibers to influence the capillarity coefficient and porosity in mixtures is well-documented, particularly with the inclusion of glass fibers. Research has shown that glass fibers improve the impermeability of concrete by reducing microcracks and porosity, thereby enhancing durability [31,45]. However, the performance of glass fiber-reinforced concrete (GFRC) can vary significantly based on the fiber content and length. For example, studies show that moderate fiber ratios enhance mechanical and durability properties, but excessive amounts can cause fiber clustering, adversely affecting performance [28]. Consequently, the careful selection of glass fiber type, ratio, and length is critical for optimizing the design of cementitious mortars [27].

These findings could serve as important guidelines for the development and optimization of fiber-reinforced mixtures in the concrete industry. However, further research and experimentation using data obtained through more research are needed to better understand the effects of fibers on the properties of mortar mixtures.

### 3.6. Ultrasonic Pulse Velocity Results

**Figure 13:**
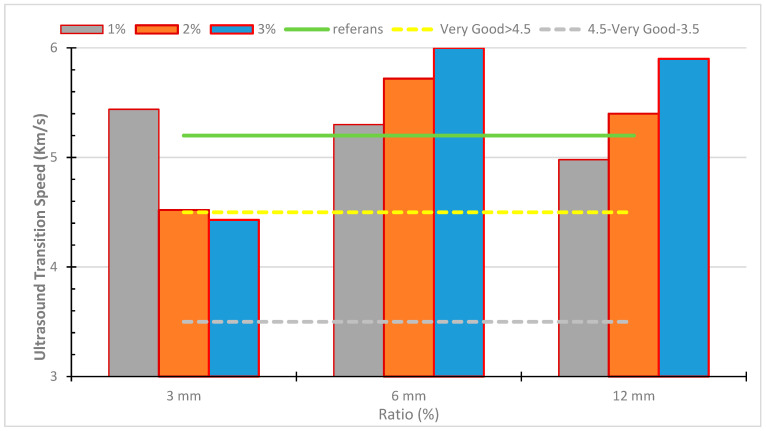
Ultrasonic pulse velocity of mortar specimens with different fiber lengths and ratios.

Figure 13 displays the measured ultrasonic pulse velocities for samples with different fiber ratios and lengths. For 3 mm fiber length, it is observed that the samples with a 1% fiber ratio have the highest pulse velocities (5.44 km/s), and as the fiber ratio increases, these values decrease. Similarly, this trend is observed for 6 mm and 12 mm fiber lengths. However, the lowest overall pulse velocity among all mixtures was observed for the 12 mm-3% mixture (5.30 km/s), while the 6 mm-1% mixture exhibited the highest value (6.00 km/s).

When compared with the pulse velocity of the reference sample (5.20 km/s), it is noticed that the measured values for most samples are higher than the reference value. This suggests that fiber addition generally increases the pulse velocities of mortar. However, in some cases, particularly for the 12 mm-3% mixture, it is observed that the measured values are only slightly above the reference value.

It should be noted that the measured UPV values (5.3–6.0 km/s) are higher than typical values reported for cement mortars, which normally range between 3.0–4.5 km/s [40,42]. This anomaly is likely attributed to the small specimen size used in this study, which may cause near-field effects. In the near-field region, the first arrival of the P-wave can be influenced by reflected waves from specimen boundaries, leading to overestimated pulse velocities [19,29]. The relatively dense mortar matrix (w/c = 0.50) and the use of standard sand may also contribute to the higher values, but the near-field effect remains the most probable explanation for the unusually high velocities. Future studies should consider using larger specimens (e.g., 100 mm cubes or prisms) to minimize near-field effects and obtain more representative UPV values [44].

The measured ultrasonic pulse velocities are evaluated according to the concrete quality classification defined by ASTM C 597 [40] standard. According to this standard, values above 4.5 km/s are classified as “excellent,” values between 3.5–4.5 km/s are classified as “good,” values between 3.0–3.5 km/s are classified as “fair,” and values below 3.0 km/s are classified as “poor.” In most samples, the measured pulse velocities (ranging from 5.30 to 6.00 km/s) fall into the “excellent” category, indicating high-quality mortar. However, it should be noted that these high values may be influenced by near-field effects due to the small specimen size, as discussed above.

### 3.7. SEM and EDS Analysis

Scanning electron microscopy (SEM) (Quanta 650 Field Emission SEM, Thermo Fisher Scientific, Eindhoven, The Netherlands) and energy-dispersive X-ray spectroscopy (EDS) analyses were performed to characterize the microstructure and chemical composition of the mortar samples. Within this scope, the reference mortar (without fibers) and specimens with varying fiber lengths (6 mm and 12 mm) and ratios (1%, 2%, and 3%) were examined.

**Figure 14:**
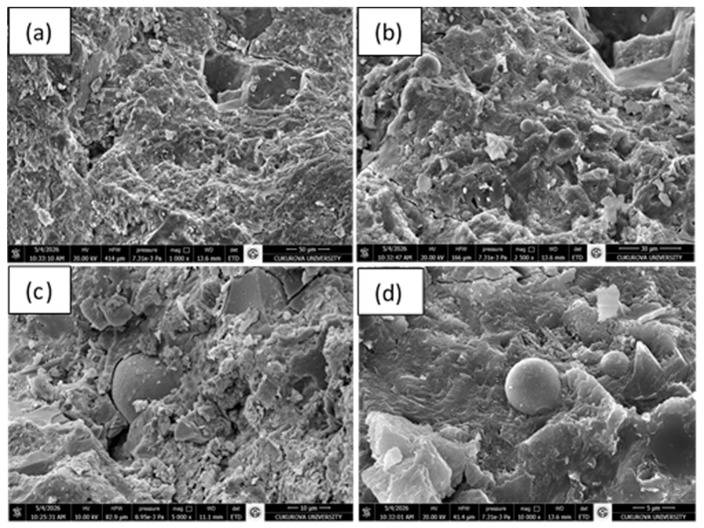
SEM images of the reference mortar (without fiber reinforcement) at different magnifications: (**a**) 1000×, (**b**) 2500×, (**c**) 5000×, and (**d**) 10,000×.

The higher magnification SEM micrographs (Figure 14c,d) show intact, smooth spherical particles embedded within the hydration products. These features are attributed to the unhydrated/partially reacted mineral components or core precursor particles within the cementitious blend. While these rigid microspheres did not undergo complete chemical dissolution during the hydration process, they contribute to the physical packing of the matrix by occupying micro-voids, which correlates with the low capillarity coefficients observed in well-consolidated mixtures [40]. It should be noted that the EDS analyses presented in this section represent localized micro-zones rather than the bulk matrix composition. Therefore, the reported Ca/Si ratios reflect the chemical heterogeneity of specific regions (such as fiber-matrix interfaces or CH-enriched zones) and should not be interpreted as the average composition of the mortar matrix.

**Figure 15:**
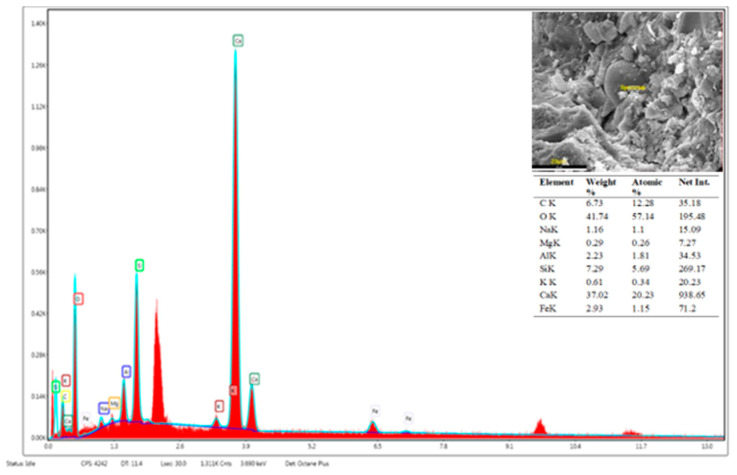
EDS analysis from reference mixture.

EDS analysis (Figure 15) confirmed the matrix composition, showing 37.02 wt.% Ca, 7.29 wt.% Si, 2.23 wt.% Al, and 6.73 wt.% C, indicating the presence of C-S-H gel, portlandite (CH), and carbonation products. The atomic Ca/Si ratio of approximately 3.55 was calculated for this localized zone, which is higher than typical C-S-H gel values (1.5–2.0), suggesting the presence of CH-rich phases in this specific micro-region. This relatively high ratio, along with the detected carbon content, points to the potential coexistence of CH and calcium carbonate (CaCO_3_) phases within the analyzed zone.

**Figure 16:**
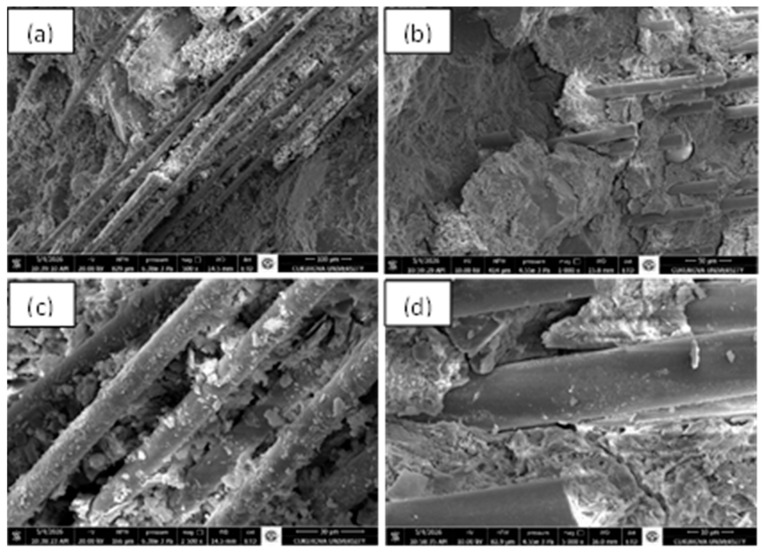
SEM images of the 6 mm-1% glass fiber-reinforced mortar: (**a**) 500×, (**b**) 1000×, (**c**) 2500×, and (**d**) 5000× magnification.

At 1% fiber content (6 mm-1%), SEM images (Figure 16) show that the glass fibers are homogeneously distributed within the cementitious matrix with no significant agglomeration. The fiber-matrix interface appears generally well-bonded, with no observable detrimental voids or microcracks. Notably, at higher magnifications (Figure 16c,d), a continuous layer of C-S-H gel along with hydration products is clearly visible on the fiber surfaces. This matrix attachment on the fiber surfaces suggests strong chemical adhesion and effective mechanical interlocking between the glass fibers and the surrounding mortar matrix [46,47].

**Figure 17:**
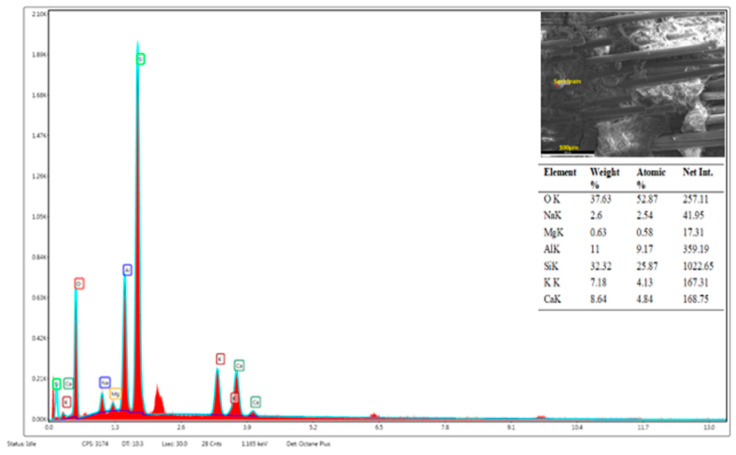
EDS analysis of the 6 mm-1% mixture.

EDS analysis (Figure 17) was focused on the matrix region situated directly beneath the glass fibers to evaluate the local interfacial chemistry. The spectrum revealed a silicon-rich and aluminum-rich profile (32.32 wt.% Si and 11.00 wt.% Al), accompanied by a low calcium content (8.64 wt.% Ca). This low calcium concentration indicates a modified hydration product distribution in the micro-environment around the fibers [48]. This variation is likely caused by the local packing effects of cement particles, which alters the chemical composition at the fiber-matrix interface [49]. The very low Ca/Si ratio (~0.27) indicates that this localized micro-area is dominated by the silica-rich glass fiber surface rather than the surrounding cementitious matrix.

**Figure 18:**
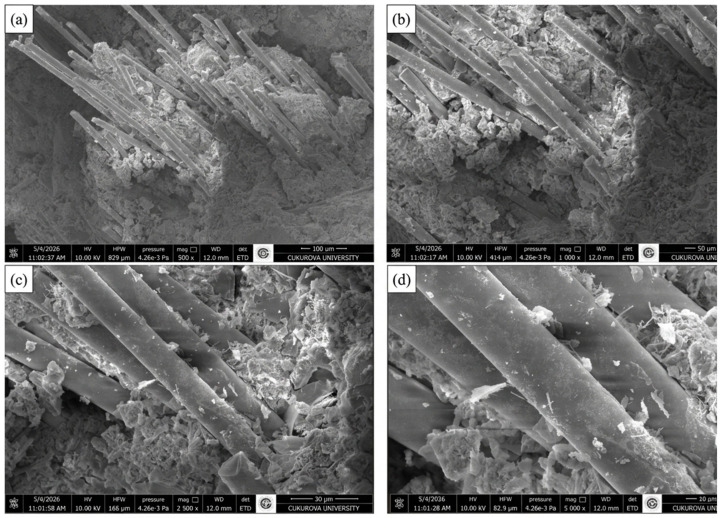
SEM images of the 6 mm-2% glass fiber-reinforced mortar: (**a**) 500×, (**b**) 1000×, (**c**) 2500×, and (**d**) 5000× magnification.

At 2% fiber content (6 mm-2%), SEM images (Figure 18) exhibit a noticeably higher fiber density within the matrix. Although the fibers remain relatively well distributed, enhanced fiber-fiber interactions and overlapping are observed compared to the 1% mixture. This increased fiber concentration leads to the formation of localized micro-voids around some fiber bundles (Figure 18a,b), likely due to the restricted penetration and compaction of the cement paste in densely reinforced regions. Furthermore, higher magnification images (Figure 18c,d) reveal several clean fiber surfaces with limited matrix adhesion, suggesting weaker interfacial bonding and a greater tendency for fiber pull-out during the fracture process.

**Figure 19:**
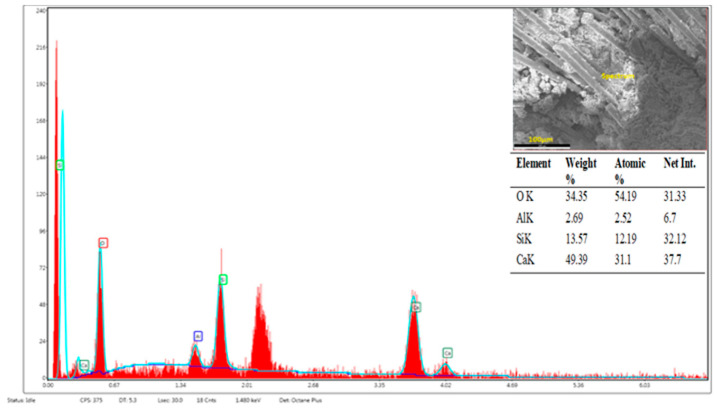
EDS analysis of the 6 mm-2% mixture.

EDS analyses (Figure 19) revealed distinct chemical zones in the micro-environment of the 2% fiber mixture, tracking areas with high localized calcium concentrations (with Ca content reaching up to 49.39 wt.%). These localized elemental profiles suggest a significant accumulation of calcium hydroxide (portlandite) crystals surrounding the fiber matrices [50]. The results indicate that the higher fiber dosage may promote the migration and accumulation of Ca^2+^ ions toward the fiber-matrix boundaries during early hydration, leading to a localized CH enrichment in the interfacial zone [51].

**Figure 20:**
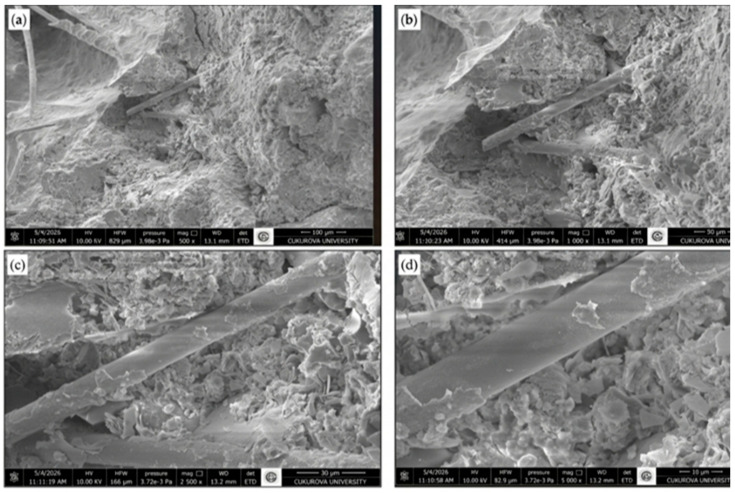
SEM images of the 6 mm-3% glass fiber-reinforced mortar: (**a**) 500×, (**b**) 1000×, (**c**) 2500×, and (**d**) 5000× magnification.

At 3% fiber content (6 mm-3%), SEM images (Figure 20) reveal a highly heterogeneous microstructure characterized by poor matrix compaction. Due to the excessively high fiber volume, pronounced fiber bundling is observed, creating restricted zones where the cement paste fails to penetrate between individual strands (Figure 20a,b). Consequently, extensive interconnected micro-voids and gaps are formed around the fiber clusters. Furthermore, high-magnification views (Figure 20c,d) show predominantly smooth and clean fiber surfaces with only limited hydration products attached, indicating weak fiber-matrix interaction and reduced interfacial bonding. These microstructural features are expected to adversely affect the mechanical performance of the composite.

**Figure 21:**
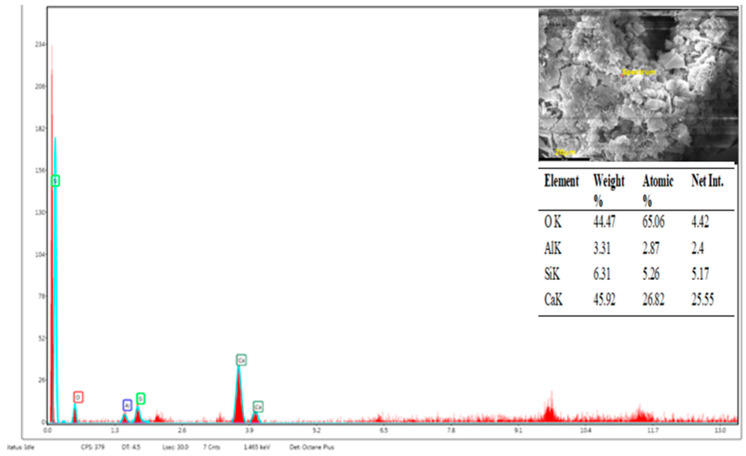
EDS analysis of the 6 mm-3% mixture.

EDS analysis of the 3% fiber-reinforced mixture (Figure 21) reveals a high localized calcium concentration, with the Ca content reaching 45.92 wt.% (26.82 at.%) and oxygen at 44.47 wt.% (65.06 at.%). This elemental distribution indicates a substantial accumulation of calcium-rich hydration products within the analyzed micro-zone. The relatively low amounts of silicon (6.31 wt.%) and aluminum (3.31 wt.%) further suggest that this region is chemically distinct from the surrounding C-S-H-rich matrix. These findings demonstrate a highly heterogeneous elemental distribution, which is consistent with the poor matrix compaction, interconnected voids, and structural discontinuities observed in the corresponding SEM micrographs of the 3% fiber- reinforced sample.

**Figure 22:**
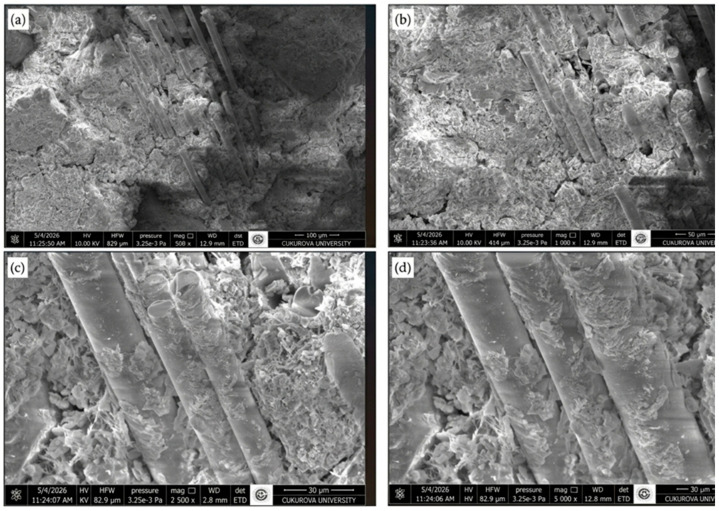
SEM images of the 12 mm-1% glass fiber-reinforced mortar: (**a**) 500× (**b**) 1000×, (**c**) 2500×, and (**d**) 5000× magnification.

At 1% fiber content (12 mm-1%), SEM images (Figure 22) show that the fibers are more curved and irregularly oriented compared to the 6 mm fibers. Fiber-matrix adhesion is asymmetric, with linear voids forming along some fiber surfaces (Figure 22c,d).

**Figure 23:**
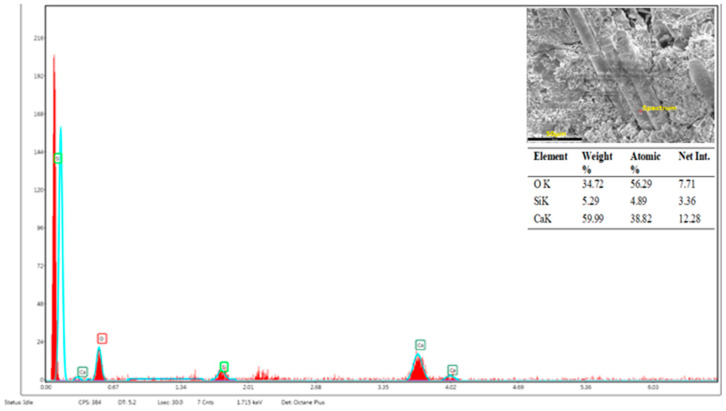
EDS analysis of the 12 mm-1% mixture.

EDS analysis (Figure 23) revealed a matrix composition primarily consisting of 59.99% Ca, 34.72% O, and 5.29% Si by weight. The high Ca content indicates a significant presence of calcium-rich phases, likely associated with CH (calcium hydroxide) accumulation within the matrix.

**Figure 24:**
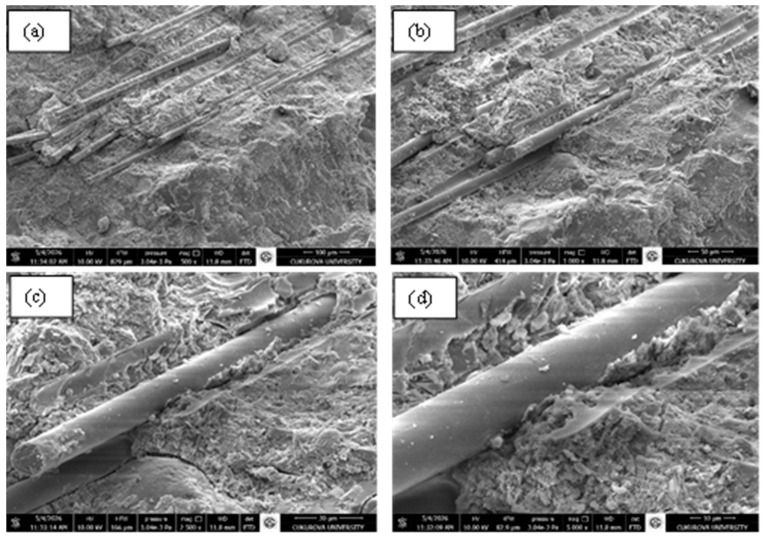
SEM images of the 12 mm-2% glass fiber-reinforced mortar: (**a**) 500×, (**b**) 1000×, (**c**) 2500×, and (**d**) 5000× magnification.

At 3% fiber content (12 mm-3%), SEM images at low magnification (Figure 24) exhibit the most severe microstructural degradation among all mixtures. Pronounced fiber bundling is observed (Figure 24a,b), accompanied by large voids and widespread matrix discontinuities throughout the cementitious matrix. Higher-magnification images (Figure 24c,d) further reveal extensive debonding at the fiber-matrix interface together with numerous microcracks in the interfacial transition zone. These microstructural defects indicate weak interfacial bonding and provide preferential paths for crack initiation and propagation, which are expected to adversely affect the mechanical performance of the composite.

**Figure 25:**
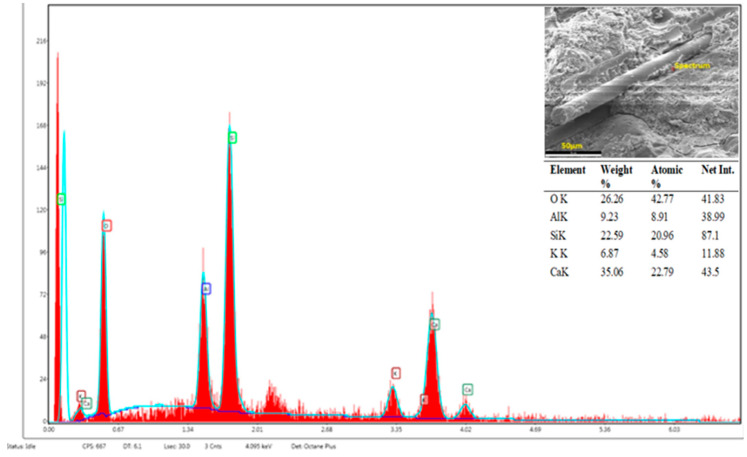
EDS analysis of the 12 mm-2% mixture.

EDS elemental analysis (Figure 25) was performed on the matrix adjacent to the bundled fibers to evaluate the local chemical composition. The microanalysis revealed a matrix composition primarily consisting of 35.06 wt.% Ca, 22.59 wt.% Si, 9.23 wt.% Al, and 6.87 wt.% K. The relatively high calcium content indicates the presence of calcium-rich hydration products within the analyzed region. However, when correlated with the SEM observations (Figure 24), the predominantly smooth fiber surfaces and limited matrix attachment suggest that these hydration products did not establish effective interfacial bonding with the glass fibers. The severe fiber bundling and the associated voids appear to have restricted the contact between the cementitious matrix and the fiber surfaces, resulting in a weak and discontinuous fiber-matrix interface.

**Figure 26:**
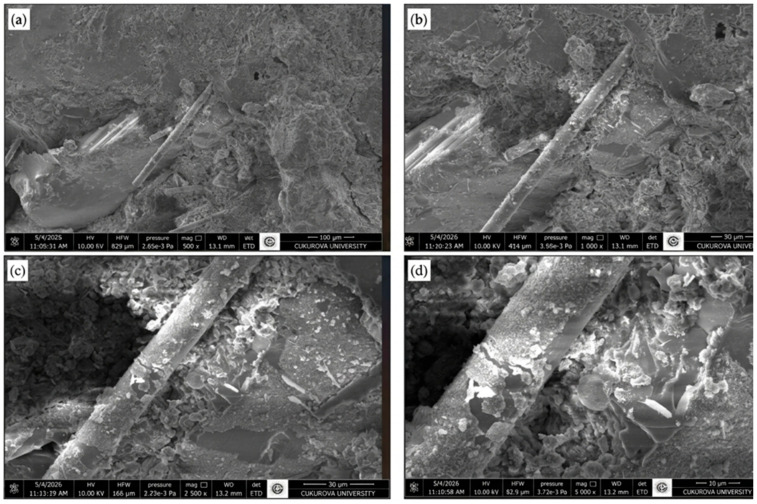
SEM images of the 12 mm-3% glass fiber-reinforced mortar: (**a**) 500×, (**b**) 1000×, (**c**) 2500×, and (**d**) 5000× magnification.

At the highest fiber content of 3% (12 mm-3%), SEM micrographs at higher magnification (Figure 26) reveal a critical level of microstructural deterioration characterized by poor matrix continuity and severe packing difficulties. As previously observed in the low-magnification images (Figure 24), the high volume of glass fibers disrupts the homogeneity of the mortar, resulting in a porous, layered, and fragmented matrix structure. Higher-magnification images (Figure 26c,d) further reveal extensive microcracks and pronounced debonding at the fiber-matrix interface. The cementitious matrix is unable to fully encapsulate the densely packed fibers, leading to limited interfacial contact and weak mechanical interlocking. Instead of forming a continuous and compact matrix, the microstructure consists of discontinuous hydration regions and interconnected voids, which are consistent with the reduced mechanical performance observed for the 3% fiber-reinforced composite.

**Figure 27:**
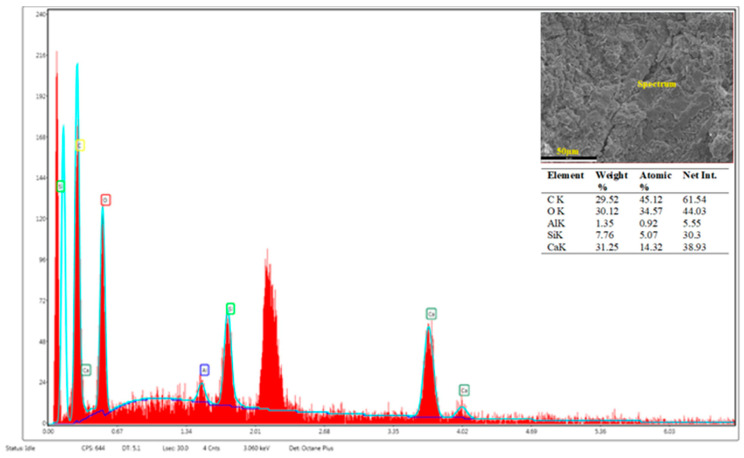
EDS analysis of the 12 mm-3% mixture.

The elemental spectrum (Figure 27) exhibits a composition predominantly comprising 31.25 wt.% Ca, 30.12 wt.% O, 29.52 wt.% C, 7.76 wt.% Si, and 1.35 wt.% Al. The relatively high calcium and carbon contents indicate the presence of calcium-rich phases within the analyzed region. In contrast, the lower concentrations of silicon and aluminum suggest that this micro-region contains fewer cementitious gel phases than the surrounding matrix, indicating a chemically heterogeneous interfacial zone. These findings are consistent with the discontinuous microstructure and interfacial defects observed in the corresponding SEM micrographs.

Overall, the SEM and EDS analyses demonstrate that both fiber content and fiber length have a significant influence on the microstructure of the mortar composites. The mixture containing 6 mm glass fibers at a dosage of 1% exhibited the most homogeneous microstructure, characterized by uniform fiber distribution, good fiber-matrix contact, and limited void formation. In contrast, increasing the fiber dosage to 3% and using 12 mm fibers resulted in pronounced fiber bundling, discontinuous matrix regions, and extensive interfacial voids. These microstructural observations are consistent with the mechanical and physical test results, which showed reductions in compressive and flexural strengths, increased water absorption and capillary water uptake, and decreased workability with increasing fiber content and fiber length [52].

### 3.8. X-Ray Diffraction (XRD) Analysis Results

**Figure 28:**
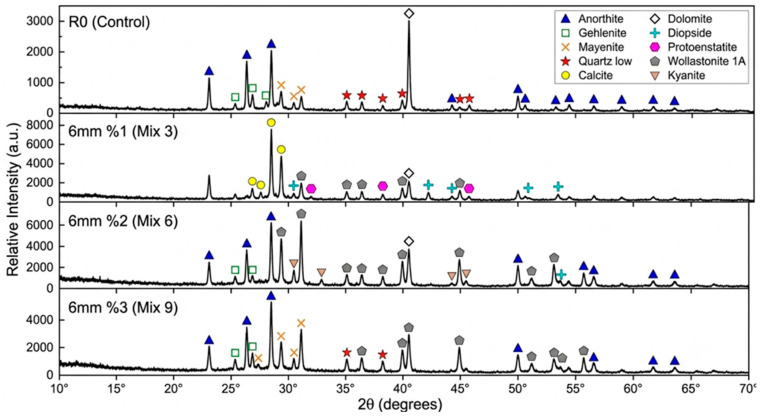
Comparative XRD patterns of 6 mm long glass fiber-reinforced mortar samples (Mix 3, Mix 6, and Mix 9) alongside the control sample (R0).

Comparative XRD analysis (Figure 28) provides robust mineralogical validation for the microstructural and chemical transformations observed in the SEM/EDS investigations. For the optimal 6 mm-1% formulation (Mix 3), a phenomenal increase in the intensity of the calcite (CaCO_3_) peak is observed at 2Ꝋ ≈ 29.5°, reaching relative intensities above 8000. This indicates a highly efficient carbonation and crystallization process within the micro-voids, directly corroborating the lowest porosity and superior fiber-matrix adhesion previously noted for this mixture [53]. As the fiber content increases to 2% Mix 6, a distinct transition towards well-crystallized wollastonite (CaCO_3_) phases becomes prominent, representing alternative calcium-silicate structures along the interfacial zones [54]. However, at the highest fiber loading of 3% Mix 9, a sharp decline in these primary reaction peaks is accompanied by the re-emergence of unreacted binder phases such as anorthite and mayenite.

This mineralogical trend indicates that the high fiber content at 3% hinders the formation of a dense cementitious matrix, which is consistent with the smooth fiber surfaces, discontinuous matrix structure, and weak fiber-matrix interaction observed in the SEM analysis.

**Figure 29:**
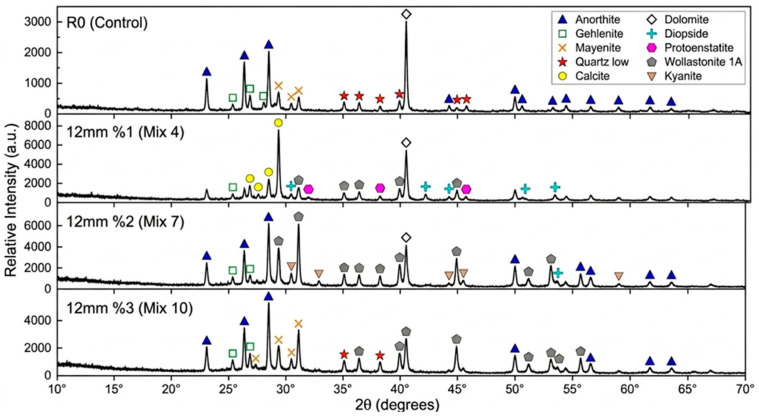
Comparative XRD patterns of 12 mm long glass fiber-reinforced samples (Mix 4, 7, 10) with control sample (R0).

Comparative XRD analysis of the 12 mm series (Figure 29) provides valuable insights into the mineralogical composition and phase evolution at different fiber contents. For the 12 mm-1% mixture (Mix 4), a prominent calcite (CaCO_3_) diffraction peak is observed at 2θ ≈ 29.5°, with an intensity exceeding 8000. This indicates a higher degree of calcite formation and is consistent with the denser microstructure observed in the corresponding SEM analysis. As the fiber content increases to 2% (Mix 7), the calcite peak intensity decreases, while diffraction peaks corresponding to wollastonite (CaSiO_3_) become more evident. However, for the 12 mm-3% mixture (Mix 10), the intensities of the primary hydration-related phases are noticeably reduced, whereas diffraction peaks associated with anorthite and mayenite become more pronounced. These mineralogical changes suggest that increasing both the fiber length and fiber content alters the hydration environment and results in a more heterogeneous matrix. This interpretation is consistent with the SEM observations, which revealed fiber bundling, discontinuous matrix regions, and extensive voids in the 12 mm-3% mixture, corresponding to the lower mechanical performance obtained for this formulation.

## 4. Conclusions

In this research, glass fibers (GFs) were incorporated into mortars at 1%, 2%, and 3% by cement weight, using three different fiber lengths (3 mm, 6 mm, and 12 mm). The main conclusions are as follows:

- Glass fiber addition progressively reduces the flow diameter due to fiber entanglement, with the most pronounced decrease (~45% reduction) occurring at 3% fiber content regardless of length.
- Dry bulk density initially increases at 1–2% fiber content but decreases at 3% dosage, with the 12 mm-3% mixture dropping below 2100 kg/m^3^ due to fiber-induced air entrapment and matrix discontinuity. The 3 mm-3% and 6 mm-3% mixtures remained above this threshold.
- The capillary water absorption coefficient remains below 0.10 mm/min^0.5^ at 1% fiber content (“good” resistance per Taywood’s criteria), but reaches 0.19 mm/min^0.5^ for the 12 mm-3% mixture, indicating severe transport degradation at high fiber loading.
- UPV results show optimal values for the 6 mm-1% mixture (6.00 km/s), while the 12 mm-3% mixture exhibits the lowest values (5.30 km/s), confirming internal structural defects and macro-porosity.
- Flexural strength at 28 days increases with higher fiber content, particularly for 6 mm fibers (up to +10.57%), owing to enhanced hydration and ITZ densification.
- Compressive strength consistently decreases with higher fiber content. The 6 mm-1% mixture achieves the highest value (60.18 MPa at 28 days), while 12 mm-3% shows the most severe reduction (−13.4% compared to 1% content).
- Microstructural analyses (SEM/EDS and XRD) confirm that a 1% fiber content promotes matrix densification through calcite crystallization within micro-voids. Conversely, a 3% fiber content leads to severe fiber bundling, with localized EDS micro-areas showing Ca contents as high as 49–65 wt.% (reflecting localized portlandite-rich deposits rather than bulk matrix composition), as well as carbonation-induced degradation facilitated by interfacial voids.

In summary, the 6 mm-1% combination provides the best overall performance: homogeneous fiber distribution, good fiber-matrix adhesion, high compressive strength, low capillarity (below 0.10 mm/min^0.5^), and enhanced flexural strength. Under the specific conditions of this study (0.50 w/c, 3 a/c, 28-day curing), the optimal fiber length is 6 mm with a recommended content of 1–2%. These findings offer practical guidance for designing fiber-reinforced mortars requiring crack control and durability. Future research should evaluate long-term performance under aggressive environmental exposure.

## Figures and Tables

**Table 1:** Physical and Chemical Properties of CEM I 42.5 R Cement.

Chemical Properties (%)		Physical Properties	
SiO_2_	21.13	Specific Gravity	3.14
Al_2_O_3_	5.27	Blaine Fineness (cm^2^/g)	3366
Fe_2_O_3_	3.23	Loss on Ignition (%)	2.47
CaO	61.98	Compressive Strength (MPa)	28.5 (2 days) 48.0 (7 days) 55.0 (28 days)
MgO	1.98		
Na_2_O	0.39	Initial Setting Time (minutes)	150
K_2_O	0.81	Final Setting Time (minutes)	210
SO_3_	3.11		
Na_2_O + 0.658 × K_2_O (%)	0.92		

**Table 2:** Grain Size Distribution of Reference Sand.

Square Sieve Opening (mm)	Cumulative Retained on Sieve (%)
2.00	0
1.60	7 ± 5
1.00	33 ± 5
0.50	67 ± 5
0.16	87 ± 5
0.08	±1

**Table 3:** Technical properties of glass fiber.

Feature	Value
Fiber Type	Glass
Fiber Length (mm)	3, 6, 12
Fiber Diameter (µm)	13
Specific Gravity (g/cm^3^)	2.68
Modulus of Elasticity (MPa)	72,000
Tensile Strength (MPa)	1700

**Table 4:** Mixture Components and Ratios.

Mix ID	Fiber Length (mm)	Fiber Ratio (%)	Cement (g)	Water (g)	Aggregate (g)	Compressive Strength ± SD (MPa)	Flexural Strength ± SD (MPa)
Ref.	-	-	450	225	1350	57.77 ± 1.28	8.50 ± 0.30
3 mm-1%	3	1	450	225	1350	59.82 ± 1.38	8.80 ± 0.33
6 mm-1%	6	1	450	225	1350	60.18 ± 1.42	9.14 ± 0.42
12 mm-1%	12	1	450	225	1350	58.98 ± 1.55	8.70 ± 0.29
3 mm-2%	3	2	450	225	1350	49.76 ± 1.90	9.34 ± 0.35
6 mm-2%	6	2	450	225	1350	57.18 ± 1.62	9.64 ± 0.38
12 mm-2%	12	2	450	225	1350	54.43 ± 1.70	9.45 ± 0.36
3 mm-3%	3	3	450	225	1350	48.74 ± 1.85	9.07 ± 0.34
6 mm-3%	6	3	450	225	1350	53.43 ± 1.68	9.43 ± 0.37
12 mm-3%	12	3	450	225	1350	49.77 ± 1.82	9.35 ± 0.36

## Data Availability

The original contributions presented in this study are included in the article. Further inquiries can be directed to the corresponding author.
